# Tumor suppressing role of serum-derived exosomal microRNA-15a in osteosarcoma cells through the GATA binding protein 2/murine double minute 2 axis and the p53 signaling pathway

**DOI:** 10.1080/21655979.2021.1987092

**Published:** 2021-10-21

**Authors:** Chunyu Wu, Zhigang Li, Guang Feng, Liqin Wang, Jingri Xie, Yang Jin, Long Wang, Songjiang Liu

**Affiliations:** a Department of Continuing Education, Affiliated Hospital of Heilongjiang University of Chinese Medicine, Harbin, Heilongjiang, P.R. China; b Department of the Second Ward Orthopedics, The Second Affiliated Hospital of Heilongjiang University of Chinese Medicine, Harbin, Heilongjiang, P.R. China; c Department of Youth League Committee, Heilongjiang University of Chinese Medicine, Harbin, Heilongjiang, P.R. China; d Department of Vice Director of the Hospital, Affiliated Hospital of Heilongjiang University of Chinese Medicine, Harbin, Heilongjiang, P.R. China; e Department of the Liver Spleen and Stomach, Affiliated Hospital of Heilongjiang University of Chinese Medicine, Harbin, Heilongjiang, P.R. China; f Department of the Graduate School, Heilongjiang University of Chinese Medicine, Harbin, Heilongjiang, P.R. China; g Department of Graduate Division, Affiliated Hospital of Heilongjiang University of Chinese Medicine, Harbin, Heilongjiang, P.R. China; h Department of Oncology, Affiliated Hospital of Heilongjiang University of Chinese Medicine, Harbin, Heilongjiang, P.R. China

**Keywords:** Osteosarcoma, miR-15a, GATA2, MDM2, p53, exosomes

## Abstract

Exosomes are emerging tools for transporting lipids, proteins, microRNAs (miRNAs), or other biomarkers for clinical purposes. They have produced widespread concern in managing human diseases, including osteosarcoma (OS). This study focuses on the function of serum-derived exosomal miR-15a in the growth of OS cells and the mechanism of action. Differentially expressed genes between OS and normal samples were screened using two datasets GSE70367 and GSE65071. miR-15a was poorly expressed, whereas GATA-binding protein 2 (GATA2) and murine double minute 2 (MDM2) were abundantly expressed in OS samples. miR-15a and its target mRNAs, including GATA2, were enriched in the p53 signaling pathway. miR-15a directly targets GATA2 mRNA to inhibit its expression, whereas GATA2 activates the transcription of MDM2, a negative regulator of p53. Overexpression of GATA2 and MDM2 promoted proliferation and cell cycle progression of MG-63 cells, whereas miR-15a blocked this axis and suppressed cell growth. miR-15a was identified as a major cargo of serum-derived exosomes, and exosomes conveying miR-15a were internalized by OS cells. This study demonstrated that miR-15a suppresses the GATA2/MDM2 axis to inhibit the proliferation and invasiveness of OS cells *in vitro* through the p53 signaling pathway.

## Introduction

Osteosarcoma (OS) is characterized by a high incidence of recurrence and metastasis, a high degree of malignancy, and poor treatment outcome. It is the most frequently diagnosed bone malignancy in children and adolescents [1]. It has been reported that the occurrence of OS is correlated with genetic factors [2]. Conventional surgical resection and chemotherapy have been the major treatments for OS intervention and improved the survival opportunity of patients; however, the 5-year survival rate of patients remains unsatisfactory, especially in those with metastatic and recurrent disease [3–5]. The complexity of the onset and progression mechanisms of OS is a major obstacle in developing novel therapeutic options and improving the survival rate of OS patients.

MicroRNAs (miRNAs) are small non-coding RNAs (approximately 22 nucleotides in length) that play a primary role in post-transcriptional regulation of gene expression by directly binding to the 3ʹ-untranslated region of mRNAs [6]. miRNAs play versatile functions in cancer development and may serve as predictive, diagnostic, and therapeutic targets/tools for human malignancies, including OS [7,8]. In this study, bioinformatic analyses using Gene Expression Omnibus (GEO) datasets, GSE70367 and GSE65071, suggested that miR-15a is a deregulated miRNA in OS samples. miR-15a, in concert with miR-16, another miRNA in the same cluster at 13q14.3 of the human genome, has been reported as a tumor suppressor in cancer tissues [9]. The inhibitory function of miR-15a has also been demonstrated in OS [10]. Circulating miRNAs may be delivered as cargo wrapped in exosomes, therefore under better protection from RNase and may serve as endogenous controls [9]. Exosomes are 50–200 nm extracellular vesicles derived from body fluids or various metabolically active cells, which primarily function in intercellular communication and are implicated in many human diseases with their transferred cargos [11,12]. This study explored whether miR-15a can be carried by exosomes and probed the correlation between miR-15a, OS development, and the molecules involved.

Our bioinformatics analyses suggested that miR-15a and its target mRNAs were enriched in the p53 signaling pathway. The p53 pathway plays an important role in maintaining genomic stability and preventing oncogenesis, the deregulation of which is closely linked to tumorigenesis [13]. p53 gets activated as a response to specific cellular stress and triggers several protective reactions, such as cell cycle arrest, senescence, and cell death [14]. The integrated analyses suggested GATA-binding protein 2 (GATA2) as a target gene that was aberrantly expressed in OS samples, and it was enriched in p53 signaling.

GATA2 is an essential factor for hematopoietic cell development, and its aberrant activation has been demonstrated in human malignancies [15–17]. This study speculated that the abnormal expression of GATA2 was possibly associated with OS development. Another member of the GATA-binding factor family, GATA4, was reported as a positive regulator of murine double minute 2 (MDM2) [18], which has been well established as a negative regulator of the p53 signaling pathway and is involved in OS progression as well [19]. Therefore, this study hypothesized that there is a similar GATA2-MDM2 interaction in OS and exosomal miR-15a may mediate the GATA2-MDM2-p53 axis. In summary, this study aims to examine whether miR-15a can be carried by exosomes and affects the behavior of OS cells by affecting the GATA2-MDM2-p53 axis.

## Materials and methods

### Patients and clinical samples

This study was approved by the Ethics Committee of Affiliated Hospital of Heilongjiang University of Chinese Medicine and performed in accordance with the guidelines of the *Declaration of Helsinki*. Informed consent was obtained from each eligible participant. A total of 31 patients with OS who were treated at Affiliated Hospital of Heilongjiang University of Chinese Medicine from July 2014 to September 2016 were recruited for this study. The enrolled patients were free of other malignancies and had no history of chemoradiotherapy. The tissue samples were collected from patients during surgery, instantly placed in liquid nitrogen, and frozen at −80°C for further use. The clinical characteristics of the patients are summarized in Table 1. In addition, peripheral samples from healthy individuals who underwent physical examination were collected. All sample-source individuals were healthy and free of any chronic or acute disease.

**Table 1.** Clinical baseline characteristics of patients with OS

Characteristics	Number
Total	31
Sex	
Male	17
Female	14
Age (years)	
≥ 20	23
< 20	8
Tumor size (cm)	
≥ 8	4
< 8	27
TNM staging	
IA–IIA	9
IIB–IV	22

OS, osteosarcoma; TNM, tumor node metastasis.

### Dataset collection and analysis

The OS-related miRNA datasets GSE70367 and GSE65071, and the mRNA dataset GSE32395 were obtained from the GEO (https://www.ncbi.nlm.nih.gov/geo) database. The GSE70367 and GSE32395 datasets were used to screen the differentially expressed miRNAs and mRNAs between OS and normal cells, respectively. The GSE65071 dataset was used to analyze the differentially expressed miRNAs between 20 OS tissues and 15 control tissues.

### Bioinformatics analyses

All datasets were analyzed using the R language program (version 3.6.3, R). The obtained GEO-sourced datasets were loaded into the edge R package (Bioconductor, USA). Differentially expressed genes were screened using |Log_2_FoldChange| > 2 and false discovery rate (FDR) < 0.01 as the screening criteria, and the corresponding heatmaps were produced using the heatmap package (version 3.6.3, R) [20]. Expression profiles of miR-15a, GATA2, and MDM2 in OS were first predicted using The Cancer Genome Atlas (TCGA) database (https://cancergenome.nih.gov). The median gene expression in patients was determined using the Survival Program (version 3.6.3, R), based on which the patients were allocated into two groups to evaluate the correlation between gene expression and prognosis of patients using the Kaplan–Meier analysis. Gene Ontology (GO) and Kyoto Encyclopedia of Genes and Genomes (KEGG) enrichment analyses were performed using the ClusterProfiler package (Bioconductor, Seattle, Washington, USA) and visualized using the Barplot Package (version 3.6.3, R). The potential target mRNAs of miR-15a were predicted using StarBase (http://starbase.sysu.edu.cn/index.php). The annotation information of the GO biological pathway and KEGG pathway data were downloaded from the corresponding GO (http://www.bioconductor.org/packages/release/data/annotation) and KEGG (https://www.kegg.jp/kegg/rest/keggapi.html) databases, respectively. The existence of miRNAs in exosomes was predicted using the EVmiRNA system (http://bioinfo.life.hust.edu.cn/EVmiRNA/#!). The promoter sequence of MDM2 was obtained from the UCSC website (https://genome.ucsc.edu/index.html). The binding relationship between miR-15 and GATA2 was predicted using the ALGEEN system (http://alggen.lsi.upc.es/cgi-bin/promo_v3/promos).

### Reverse transcription quantitative polymerase chain reaction (RT-qPCR)

Total RNA was extracted from tissues and cells using the TRIzol reagent (Invitrogen, Inc., Carlsbad, CA, USA). The RNA was reverse transcribed into complementary DNA (cDNA) using Primescript RT reagent (Takara Holdings Inc., Kyoto, Japan). Thereafter, qPCR was performed on a StepOne Plus Real-Time PCR System (Applied Biosystems, Inc., Carlsbad, CA, USA) using the SYBR Premix Ex Taq reagent (Takara). The primer sequences are listed in Table 2, in which U6 and GAPDH were used as internal references. RT-qPCR results were measured using the 2^−ΔΔCt^ method.

**Table 2.** Primer sequences for RT-qPCR

Gene	Primer sequence (5ʹ-3ʹ)
miR-15a	F: TAGCAGCACATAATGG
	R: GTGCAGGGTCCGAGGT
GATA2	F: GCCGGGAGTGTGTCAACTG
	R: AGGTGGTGGTTGTCGTCTGA
MDM2	F: CGGTCAAGTTGGGACACGTCCACACTGG
	R: AATTCGGACGTGTCCCAACTTGACCAGTG
U6	F: CTCGCTTCGGCAGCACA
	R: AACGCTTCACGAATTTGCGT
GAPDH	F: ATCACTGCCACCCAGAAGAC
	R: TTTCTAGACGGCAGGTCAGG

RT-qPCR, reverse transcription-quantitative polymerase chain reaction; miR, microRNA; GATA2, GATA binding protein 2; MDM2, murine double minute 2; GAPDH, glyceraldehyde-3-phosphate dehydrogenase.

### Cell culture and transfection

Normal osteoblast cells (hFOB1.19) and OS cell lines (SaoS-2, MG-63, U2-OS, and HOS) were purchased from American Type Culture Collection (ATCC, Manassas, VA, USA). The cells were cultivated in Roswell Park Memorial Institute 1640 (RPMI-1640, HyClone, Logan, UT, USA) supplemented with 10% fetal bovine serum (FBS) and 100 μg/mL penicillin/streptomycin at 37°C with 5% CO_2_ [21]. Once reaching 70–80% confluence, the cells were transfected with miR-15a mimic, miR-15a inhibitor, overexpression vector (OE) of GATA2 (GATA2-OE) and MDM2-OE (all purchased from GenePharma Co., Ltd., Shanghai, China, and the pcDNA vectors were diluted in Opti-MEM). Cells transfected with pcDNA3.1 (+) empty vectors (Addgene, Cambridge, MA, USA) were used as controls [22].

### Colony formation assay

After transfection, cell proliferation was assessed. MG-63 and U2-OS cells were seeded in 6-well plates at 600 cells per well and then incubated at 37°C with 5% CO_2_. After 16 days, the cells were fixed with 4% paraformaldehyde (1 mL/well) for 30 min and stained with 500 μL Giemsa solution (Solarbio Science & Technology Co., Ltd., Beijing, China) for 15 min. The number of formed colonies (> 50 cells) was observed under a fluorescence microscope (IX71, Olympus Corporation, Tokyo, Japan) at ×100 magnification and calculated using Image J software (Version 1.7.9, National Institutes of Health, Bethesda, Maryland, USA).

### Wound-healing assay

Transfected cells were sorted in 6-well plates at a density of 5 × 10^5^ cells per well. After 12 h of cell culture, the medium was discarded and scratches were produced on the monolayer cells at 0.5–1 cm interval. Thereafter, 5% FBS-supplemented medium was added to each well, and the cells were cultured in a 37°C incubator containing 5% CO_2_ for 24 h. The width of the scratches was determined using ImageJ software. The wound healing rate of cells was determined as a reflection of the migration ability of cells [23].

### Transwell assay

The invasion ability of the cells was determined using the transwell assay. Transfected cells were resuspended in serum-free medium at a density of 1.0 × 10^5^ cells/mL. Transwell chambers (BD Biosciences, San Jose, CA, USA) were inserted into 24-well plates. The apical chambers were precoated with Matrigel (BD Biosciences) and loaded with 200 μL of cell suspension, whereas the basolateral chambers were filled with 500 μL of 10% FBS-supplemented medium. The cells were incubated at 37°C with 5% CO_2_ for 48 h. Thereafter, the cells invading the basolateral chambers were fixed for 20 min and then stained with 0.1% crystal violet for 20 min. The number of cells was calculated under a microscope with four random fields selected [24].

### Tumorigenesis in vivo

Male BALB/c nude mice (n = 24, 4 weeks old) acquired from Vital River Laboratory Animal Technology Co., Ltd. (Beijing, China) were used for the animal experiments. MG-63 and U2-OS cells stably transfected with miR-15a mimic or miR-15a inhibitor were implanted into nude mice through hypodermic injection (n = 6 for each implantation). Thereafter, the volume of xenograft tumors was determined weekly. After 4 weeks, the animals were sacrificed by intraperitoneal injection of 150 mg/kg pentobarbital sodium, after which the tumors were collected and weighed. The animal experimental protocol was approved by the Committee on the Ethics of Animal Experiments of the Affiliated Hospital of Heilongjiang University of Chinese Medicine. Significant attempts have been made to reduce the suffering of animals [25].

### Flow cytometry

For apoptosis detection, an Annexin V-fluorescein isothiocyanate (FITC)/propidium iodide (PI) kit (BD Biosciences) was used according to the manufacturer’s instructions. Briefly, the transfected cells were first incubated with FITC-conjugated Annexin V at 22°C in the dark for 15 min, and then with PI for 10 min. The samples were analyzed on a flow cytometer (Guava easyCyte HT, EMD Millipore, Billerica, MA), and the apoptosis rate of cells was evaluated using ModFit LT 3.3 software (Verity Software House, Topsham, ME, USA).

For cell cycle detection, transfected cells were sorted in 6-mm culture dishes for 72 h and then cleared in 70% ethanol for 1 h. Thereafter, the cells were washed and incubated with staining buffer (containing 10 µg/mL RNase and PI, Solarbio) at room temperature for 10 min. The cell cycle distribution was determined using the flow cytometer and ModFit LT 3.3 software.

### Western blot analysis

The cells were lysed in 2× cell lysis buffer (Beyotime Biotechnology Co., Ltd., Shanghai, China) for 15 min to obtain total protein. Protein concentration was measured using a bicinchoninic acid kit (Beyotime). Thereafter, an equal volume of protein (20 µg) was separated using 10% sodium dodecyl sulfate (SDS)-polyacrylamide gel electrophoresis and transferred to polyvinylidene fluoride membranes (EMD Millipore). The membranes were blocked with 5% (v/v) skimmed milk for 1 h and incubated with primary antibodies at 4°C for 16 h. The antibodies used were Bcl-2 (ab692, 1:500, Abcam Inc., Cambridge, MA, USA), Bax (ab32503, 1:2,000, Abcam), GAPDH [(#3683, 1:1,000, Cell Signaling Technology (CST), Beverly, MA, USA)], cyclin E1 (ab33911, 1:2,000, Abcam), cyclin-dependent kinase 2 (CDK-2, ab32147, 1:1,000, Abcam), CDK-4 (ab199728, 1:2,000, Abcam), cyclin D1 (ab226977, 1:2,000, Abcam), p53 (sc-126, 1:500, Santa Cruz Biotechnology, Santa Cruz, CA, USA), tumor susceptibility gene 101 (TSG101, sc-7964, 1:1,000, Santa Cruz Biotechnology), and CD81 (sc-23,962, 1:500, Santa Cruz Biotechnology). The membranes were further incubated with HRP-labeled secondary antibody (#7074, 1:2,000, CST) at room temperature for 2 h. Thereafter, membranes were developed using enhanced chemiluminescence western blotting substrate (Thermo Fisher Scientific Inc., Waltham, MA, USA), and protein quantification was performed using Image J software [26].

### Extraction of the exosomes from serum

Peripheral blood samples from healthy individuals were collected, and the blood samples were centrifuged at 300 × *g*, 1,200 × *g*, and 10,000 × *g* at 4°C for 10, 20, and 30 min, respectively, to obtain the serum samples. The samples were further centrifuged at 100,000 × *g* at 4°C for 70 min, using a F50L-2461.5 T centrifuge (Thermo Fisher Scientific). The collected exosomes were stained with 1% uranyl acetate (pH = 4.0), and the shape of the particles was observed using a 200 kV transmission electron microscope (TEM, Titan G2 80–200 Chemistem, FEI, USA). The levels of exosome-specific biomarkers TSG101 and CD81 were determined by western blot analysis. The particles were further validated using a nanoparticle tracking analysis (NTA) system (NTA 3.2 Dev Build 3.2.16, Malvern Panalytical Ltd., UK). The Brownian motion of the particles was captured by laser beams and recorded by a camera. The peak diameter of the particles was then evaluated using the Stokes-Einstein equation (PMID: 32,060,049).

### Exosome labeling for cell treatment

The extracted exosomes were labeled using a PKH-67 fluorescence labeling kit (Sigma-Aldrich Chemical Company, St Louis, MO, USA) according to the manufacturer’s protocol. Briefly, the exosomes were resuspended in 200 μL of phosphate-buffered saline (PBS), which was mixed with 500 μL of diluent. A 1.5-mL Eppendorf tube was loaded with 4 μL of PKH-67 and 500 of μL diluent. The above solutions were mixed in the tube in the dark, and 1 mL of 10% bovine serum albumin was added to terminate the staining. MG-63 and U2-OS cells were cultured in 12-well plates until they reached 70% confluence, and the medium was replaced with fresh medium containing PKH-labeled exosomes. After a 12-h incubation, the cells were washed with PBS, fixed in 4% paraformaldehyde for 20 min, and then observed under an IX71 microscope to observe the absorption of exosomes.

### Dual-luciferase reporter gene assays

The binding sequence between miR-15a and GATA2 was predicted using StarBase (http://starbase.sysu.edu.cn). The wild-type (WT) sequence containing the putative binding site with miR-15a was inserted into pGL3 luciferase reporter vectors (Promega, Fitchburg, WI, USA) to design pGL3-GATA2-WT vectors, and the corresponding mutant type (MT) vector based on a mutant binding sequence with miR-15a was constructed, termed pGL3-GATA2-MT. HEK293T cells purchased from ATCC were sorted in 24-well plates at a density of 8 × 10^4^ cells per well. Then, the constructed vectors were co-transfected with miR-15a inhibitor or miR-15a control (empty vector) into HEK293T cells. After 48 h, the luciferase activity in cells was determined using a dual-luciferase reporter assay system (Promega, Madison, WI, USA).

### Chromatin immunoprecipitation (ChIP) assay

The OS cells were washed and lysed in SDS lysis buffer and protease inhibitor compounds (Thermo Fisher Scientific) on ice for 30 min. Thereafter, the samples were centrifuged at 14,000 × *g* at 4°C for 15 min, and incubated with anti-GATA2 (sc-267,1:500, Santa Cruz Biotechnology) (anti-IgG was used as control) at 4°C overnight. The mixture was further incubated with MDM2 chromatin fragment-contained ProteinA agarose at 4°C for 2 h to conjugate protein A and antibodies. After immunoprecipitation, the precipitates were collected after centrifugation at 4°C for 3 min. The magnetic beads were washed in PBS and added to SDS loading buffer. GATA2 expression in the RNA-protein compounds was determined by RT-qPCR.

### Statistical analysis

Data were analyzed using GraphPad Prism (version 6.0; GraphPad Software, CA). At least three independent experiments were conducted, and the data were presented as the mean ± standard deviation (SD). Differences were analyzed by the *t-test* (between two groups) and one or two-way analysis of variance (ANOVA, over two groups), followed by Tukey’s post hoc test. The survival rate of patients was determined using the Kaplan–Meier analysis. The correlation between gene expression was determined by Pearson’s correlation analysis. The log-rank test was used for statistical analysis. *p < 0.05 represents significant difference.

## Results

### Brief introduction

Integrated bioinformatics analyses indicated that miR-15a, an exosomal miRNA, is a poorly expressed miRNA in OS, and its downstream targets, including GATA2, were enriched in the p53 signaling pathway. The authors hypothesized that miR-15a from exosomes might affect the GATA2-MDM2 axis to mediate the p53 signaling pathway, consequently mediating the activity of OS. To validate this, exosomes from serum samples were collected, and the expression of miR-15a was examined. The interactions among miR-15a, GATA2, and MDM2 were validated, and altered expression of miR-15a, GATA2, and MDM2 was introduced in OS cells to explore the functions of these molecules in the cell behavior and in the activity of the p53 signaling pathway.

### Poor miR-15a expression indicates an unfavorable survival rate in patients with OS

**Figure 1.**
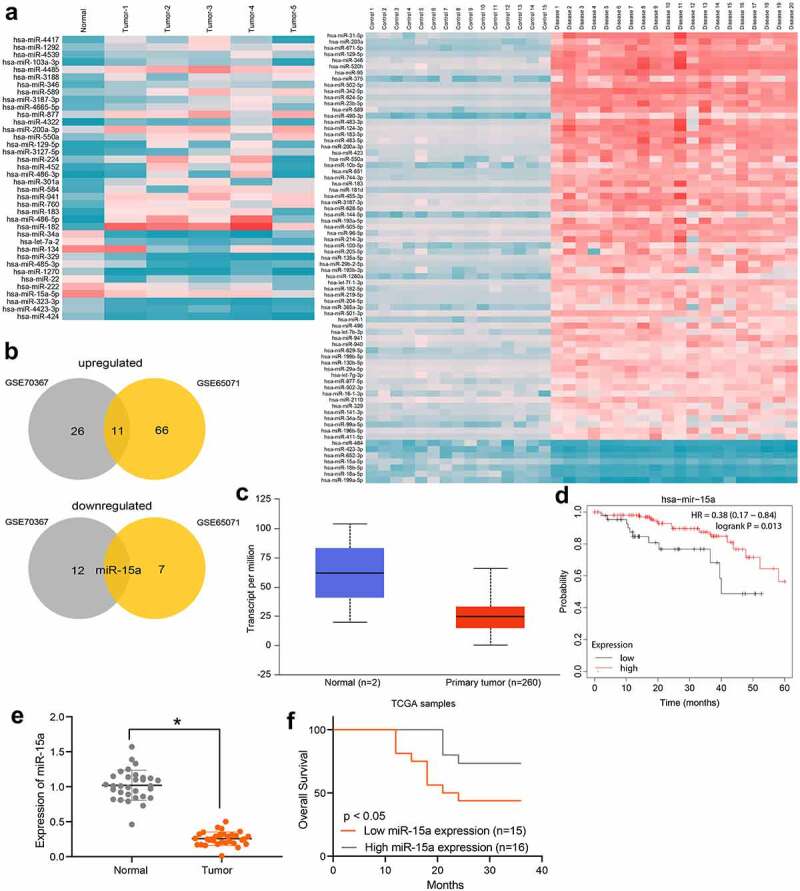
Poor miR-15a expression indicates an unfavorable survival rate in patients with OS. a, a heatmap for differentially expressed miRNAs between OS and normal samples in GEO GSE70367 and GSE65071 datasets; b, Venn diagrams for the intersections of the differentially expressed miRNAs obtained from two datasets; c, miR-15a expression in OS and normal tissues predicted on the TCGA database (*p* < 0.05, unpaired *t* test); d, the relevance between miR-15a and survival rate of patients predicted on the TCGA database (*p* < 0.05, Kaplan-Meier analysis); e, miR-15a expression in the collected tumor tissues and paired normal ones from patients determined by RT-qPCR (n = 31, *p* < 0.05, paired *t* test); f, relevance between miR-15a expression and the survival rate of patients (*p* < 0.05, Kaplan-Meier analysis). Repetition = 3

First, using |Log_2_FoldChange| > 2 and FDR < 0.01 as the criteria, differentially expressed miRNAs between OS and normal samples were screened out according to the data available from the GSE70367 and GSE65071 datasets. A heatmap is shown in Figure 1(a). The results were intersected and analyzed using a Venn diagram, and miR-15a was found to be the only miRNA downregulated in OS patients in both datasets, whereas many miRNAs were upregulated in these two datasets (Figure 1(b)). Therefore, miR-15a was selected as the study subject. We obtained data concerning miR-15a expression in OS patients and healthy individuals from TCGA database (Figure 1(c)), which suggested that miR-15a expression was lower than normal in patients with OS. Furthermore, we analyzed the correlation between miR-15a expression and the prognosis of patients in TCGA (Figure 1(d)), which suggested that patients with lower miR-15a expression showed a poorer survival rate. To validate these predicted results, we first determined miR-15a expression in patients using RT-qPCR. It was found that miR-15a expression was significantly decreased in tumor tissues compared to paired normal tissues from OS patients (Figure 1(e)). In addition, miR-15a expression in patients showed a good prognostic value. In contrast, patients with high miR-15a expression showed an increased survival rate (Figure 1(f)).

### miR-15a suppresses viability of OS cells

**Figure 2.**
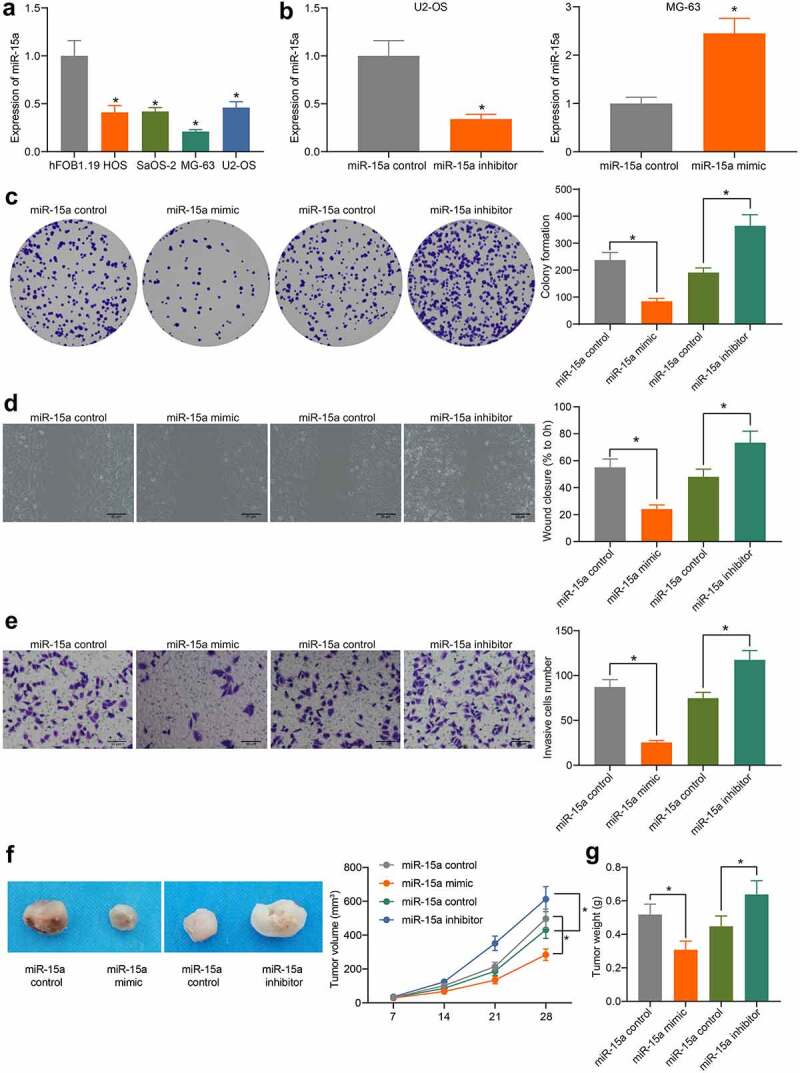
miR-15a suppresses viability of OS cells. a, miR-15a expression in OS cell lines (HOS, SaOS-2, MG-63 and U2-OS) and in normal osteoblast cells hFOB1.19 determined by RT-qPCR (*p < 0.05, one-way ANOVA); b, transfection efficacy of miR-15a mimic and miR-15a inhibitor in MG-63 and U2-OS cells, respectively, determined by RT-qPCR (*p < 0.05, unpaired *t* test); c, viability of cells determined by colony formation assay (*p < 0.05, one-way ANOVA); D-E, migration and invasion abilities of cells determined by wound-healing (d) and Transwell assay (e), respectively (*p < 0.05, one-way ANOVA); f, volume of the xenograft tumors in nude mice (*p < 0.05, two-way ANOVA); g, weight of the xenograft tumors in nude mice (*p < 0.05, one-way ANOVA). n = 6 in each group; Repetition = 3

The above findings prompted us to focus on the potential function of miR-15a in OS cells. First, miR-15a expression in OS cell lines (HOS, SaOS-2, MG-63, and U2-OS) and normal osteoblast cells, hFOB1.19, was determined by RT-qPCR. The expression of miR-15a in all OS cell lines was lower than that in Hfob1.19 cells (Figure 2(a)). Among the OS cells, MG-63 cells showed the lowest level of miR-15a, whereas U2-OS cells had the highest level, and these two cell lines were selected for subsequent experiments.

Upregulation of miR-15a was introduced in MG-63 cells, whereas downregulation of miR-15a was introduced in U2-OS cells by transfecting miR-15a mimic or inhibitor, respectively. The transfection efficacy was determined by RT-qPCR (Figure 2(b)). Thereafter, we first determined the viability of cells using the colony formation assay (Figure 2(c)), which showed that the number of colonies formed by MG-63 cells was decreased, but that of U2-OS cells was increased. In addition, according to the wound-healing and Transwell assays, the migratory and invasive potentials of MG-63 cells were reduced upon miR-15a upregulation, whereas those of U2-OS cells were increased upon miR-15a depletion (Figure 2(d,e)). To validate whether miR-15a has a similar function *in vivo*, MG-63 and U2-OS cells stably transfected with miR-15a mimic or inhibitor were implanted into nude mice. As shown in Figure 2(f), the growth of xenograft tumors in mice was reduced by miR-15a upregulation, whereas that in mice induced by U2-OS cells where miR-15a was downregulated, was increased. The mice were euthanized on the 4^th^ week, and the xenograft tumors were collected and weighed (Figure 2(g)). It was found that the weight of xenograft tumors in mice was reduced when miR-15a was upregulated, whereas the weight of tumors was increased upon miR-15a depletion.

### miR-15a promotes apoptosis and cell cycle arrests of OS cells

**Figure 3.**
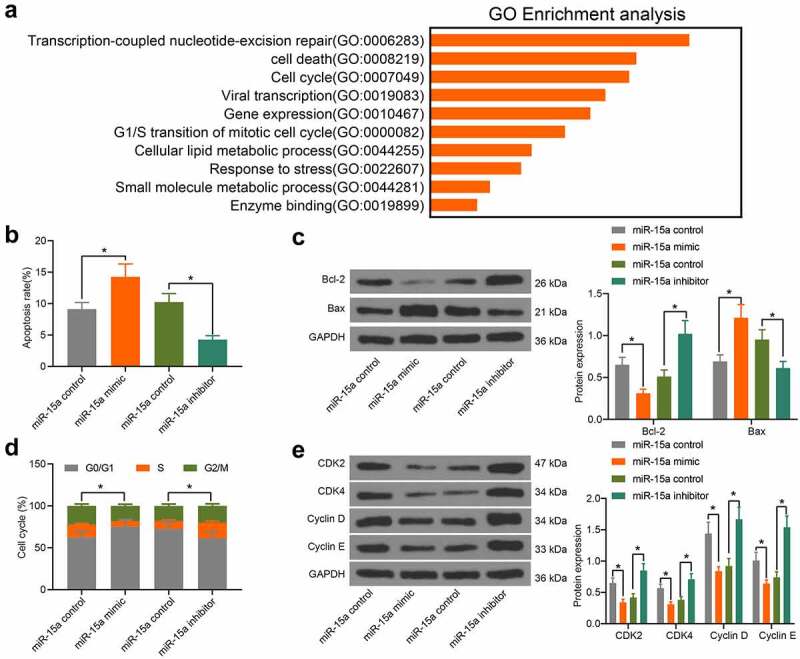
miR-15a promotes apoptosis and cell cycle arrests of OS cells. a, a GO biological enrichment analysis based on the predicted target mRNAs of miR-15a; b, apoptosis of MG-63 and U2-OS cells determined by flow cytometry (*p < 0.05, one-way ANOVA); c, protein levels of Bcl-2 and Bax in cells determined by western blot analysis (*p < 0.05, two-way ANOVA); d, cell cycle progression in cells determined by flow cytometry (*p < 0.05, two-way ANOVA); E, protein levels of CDK2, CDK4, cyclin D, cyclin E in cells determined by western blot analysis (*p < 0.05, two-way ANOVA). Repetition = 3

To further explore the influence of miR-15a on OS cell behavior, we probed the targeting mRNAs of miR-15a on Starbase. A GO biological enrichment analysis was performed based on the predicted genes using the Biological Process (BP) database. It was found that the target genes of miR-15a were mainly involved in the process of cell cycle progression and apoptosis (Figure3(a)). Therefore, we further determined the function of miR-15a in the apoptosis and cell cycle progression of transfected cells. According to flow cytometry, upregulation of miR-15a in MG-63 cells led to an increase in cell apoptosis (PI-Annexin V-positive cells), whereas downregulation of miR-15a decreased the number of apoptotic U2-OS cells (Figure 3(b)). Consistently, western blot analysis suggested that the ratio of Bax/Bcl-2 in MG-63 cells was increased, whereas that in U2-OS cells was decreased (Figure 3(c)). Flow cytometry analysis of cell cycle distribution in cells suggested that overexpression of miR-15a in MG-63 cells blocked cell cycle progression at the G0/G1 phase. Downregulation of miR-15a in U2-OS cells led to the opposite trend (Figure 3(d)). Further, western blot analysis suggested that the protein levels of CDK2, CDK4, cyclin D, and cyclin E in MG-63 cells were inhibited by miR-15a, and those in U2-OS cells were increased upon miR-15a downregulation (Figure 3e).

### miR-15a from serum-derived exosomes can be internalized by OS cells

**Figure 4.**
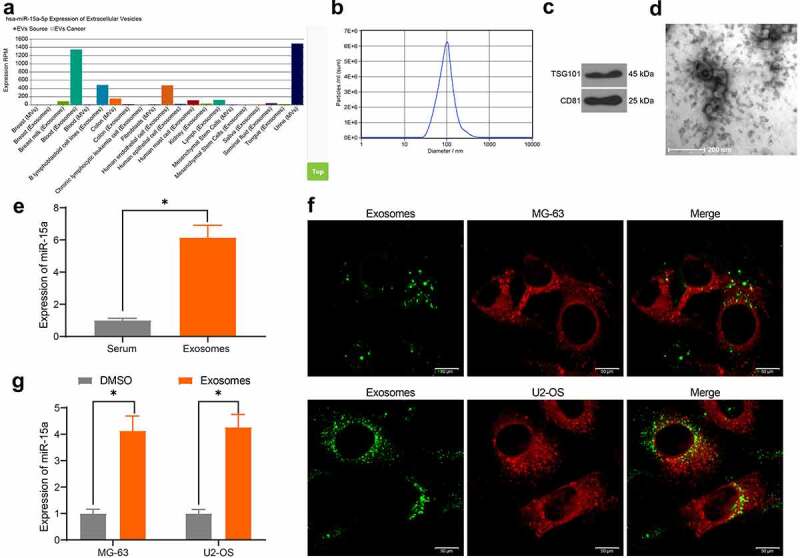
miR-15a from serum-derived exosomes can be internalized by OS cells. a, existence of miR-15a in exosomes predicted using the EVmiRNA system; b, diameter of the extracted particles evaluated by NTA; c, protein levels of TSG101 and CD81 in the particles determined by RT-qPCR; d, morphology of the particles observed under a TEM; e, miR-15a in extracted exosomes examined by RT-qPCR; f, internalization of the exosomes by OS cells confirmed by fluorescence tracking; g, miR-15a expression in OS cells after serum-derived exosome treatment determined by RT-qPCR (*p < 0.05, two-way ANOVA)

miR-15a was predicted to be a candidate cargo for blood-derived exosomes (Figure 4(a)). To validate this, we extracted exosomes from serum samples of healthy individuals using ultra-centrifugation. The NTA suggested that the average peak value of the diameter of the particles was approximately 80–150 nm, which met the standard definition of exosomes (Figure 4(b)). Western blot analysis further validated the significant positive expression of exosome biomarker proteins TSG101 and CD81 in the particles (Figure 4(c)). In addition, under TEM observation, the extracted particles presented as transparent oval-shaped bodies (Figure 4(d)). Importantly, RT-qPCR results indicated that miR-15a was highly expressed in isolated exosomes (Figure 4(e)). Next, to determine whether the exosomes could be internalized by OS cells, the exosomes were labeled with PKH-67 and co-cultured with the OS cells for 48 h. Thereafter, it was observed by fluorescence microscopy that the exosomes were absorbed by the OS cells (Figure 4(f)). In this setting, it was found that miR-15a expression in cells was significantly elevated (Figure 4(g)). Collectively, these results indicate that serum-derived exosomes can be internalized by OS cells and elevate miR-15a expression in cells.

### miR-15a directly binds to GATA2

**Figure 5.**
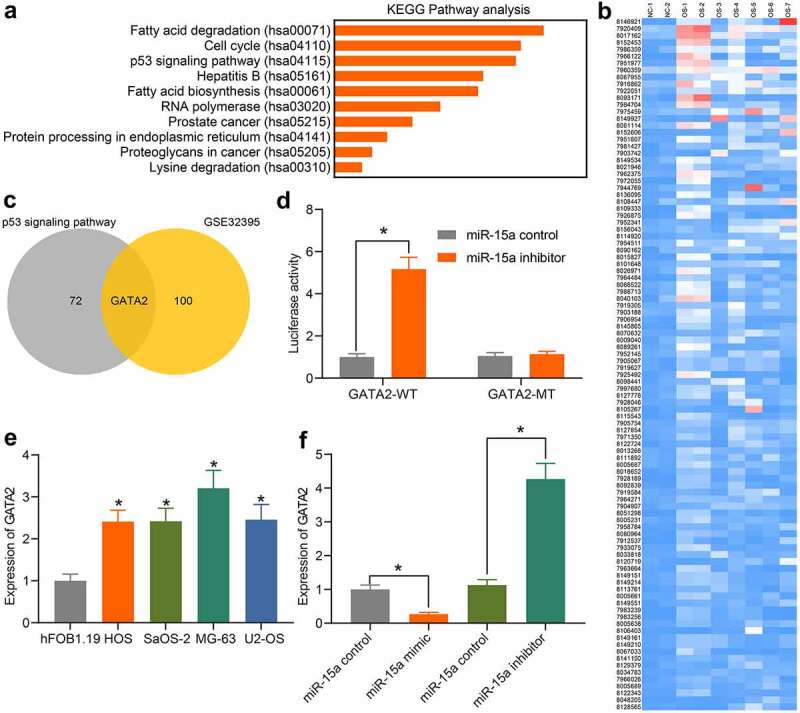
miR-15a directly binds to GATA2. a, a KEGG pathway enrichment analysis based on the predicted target mRNAs of miR-15a; b, a heatmap for differentially expressed mRNAs between normal and OS samples on the GEO GSE32395 dataset; c, a Venn diagram for the intersection of predicted mRNAs of miR-15a and the screened out differentially expressed mRNAs; d, binding relationship between miR-15a and GATA2 validated through a dual luciferase reporter gene assay (*p < 0.05, two-way ANOVA); E, GATA2 expression in OS cell lines and hFOB1.19 cells determined by RT-qPCR (*p < 0.05, one-way ANOVA); F, GATA2 expression in MG-63 and US-OS after miR-15a alteration determined by RT-qPCR (*p < 0.05, one-way ANOVA). Repetition = 3

An additional KEGG pathway enrichment analysis was performed based on the predicted target mRNAs of miR-15a (Figure 5(a)), and the p53 signaling pathway was identified as the only pathway in which the miR-15a target genes were enriched, indicating that miR-15a possibly mediates the p53 signaling pathway. To confirm the potential target mRNA of miR-15a involved in OS development, the differentially expressed mRNAs between OS and normal samples were screened using the GEO GSE32395 dataset (Figure 5(b)). An intersection analysis between the predicted mRNAs of miR-15a (those enriched in the p53 signaling pathway) and the screened mRNAs was performed, and GATA2 was found to be intersected (Figure 5(c)). Thereafter, the binding relationship between miR-15a and GATA2 was validated through a luciferase reporter gene assay (Figure 5(d)), suggesting that co-transfection of miR-15a inhibitor and pGL3-GATA2-WT in cells led to increased luciferase activity. GATA2 expression in the cells was also determined. The RT-qPCR results showed that the mRNA expression of GATA2 in OS cells was notably higher than that in hFOB1.19 cells (Figure 5(e)). In addition, the expression of GATA2 in transfected MG-63 and U2-OS cells was determined. GATA2 expression was found to be decreased after miR-15a upregulation and increased upon miR-15a downregulation (Figure 5(f)). These results indicate that GATA2 is a target gene of miR-15a, which is possibly responsible for OS development.

### GATA2 may serve as a prognostic marker indicating poor prognosis in patients

**Figure 6.**
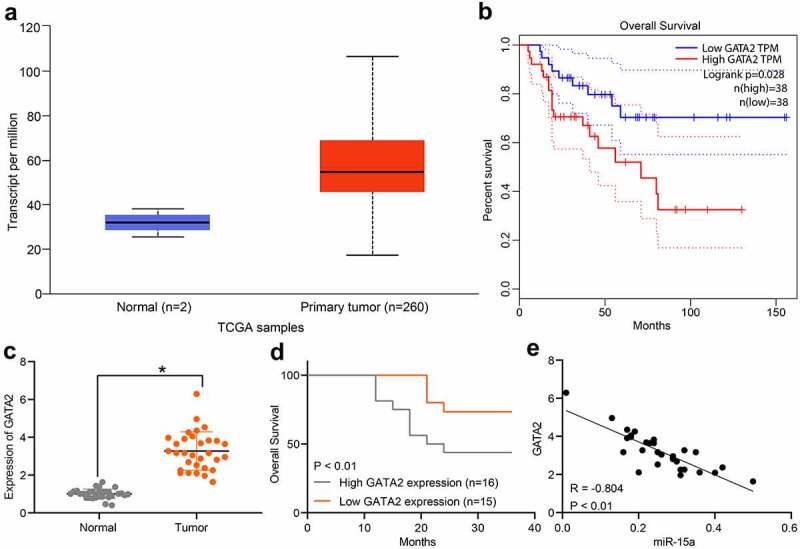
GATA2 may serve as a prognostic marker indicating poor prognosis in patients. a, GATA2 expression in OS and normal tissues predicted on TCGA database (*p* < 0.05, unpaired *t* test); b, the relevance between GATA2 and survival rate of patients predicted on TCGA database (*p* < 0.05, Kaplan-Meier analysis); c, mRNA expression of GATA2 in the collected tumor tissues and paired normal ones from patients determined by RT-qPCR (n = 31, *p* < 0.05, paired *t* test); d, relevance between GATA2 expression and the survival rate of patients (*p* < 0.05, Kaplan-Meier analysis). e, a negative correlation between GATA2 and miR-15a expression in the OS tumors (*p < 0.01, r = −0.804, Pearson’s correlation analysis). Repetition = 3

Based on the results, we further predicted GATA2 expression in OS and normal myeloid tissues in TCGA database. It was suggested that GATA2 expression was significantly increased in OS tissues (Figure 6(a)), whereas high GATA2 expression indicated a poor prognosis in patients (Figure 6(b)). To validate this, we explored GATA2 expression in the collected OS and normal samples from patients. Consequently, it was found that GATA2 was significantly upregulated in OS tissues compared to paired normal tissues from patients (Figure 6(c)). Patients with high GATA2 expression also showed a decreased survival rate (Figure6d). A negative correlation between GATA2 and miR-15a was confirmed in OS tissues from patients (Figure 6(e)).

### GATA2 mediates transcription activity of MDM2 to suppress the p53 signaling pathway

**Figure 7.**
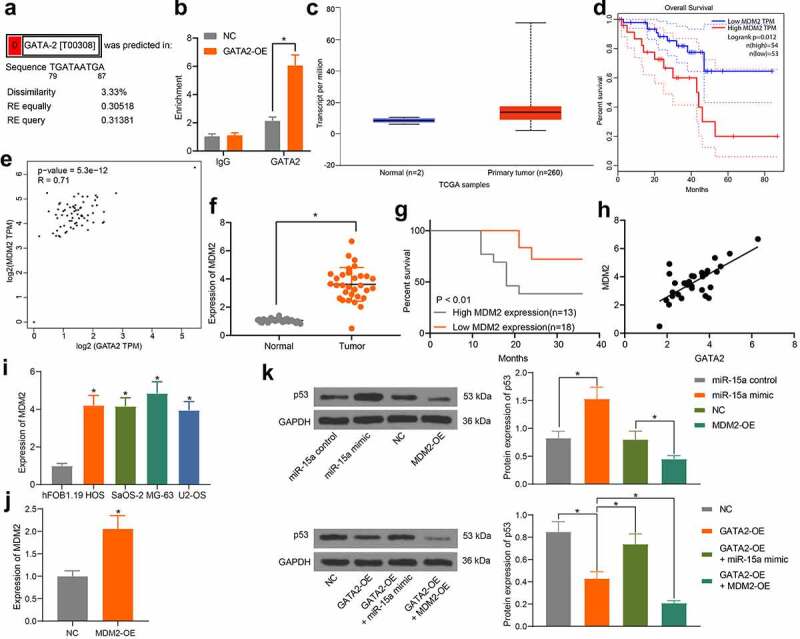
GATA2 mediates transcription activity of MDM2 to suppress the p53 signaling pathway. a, putative binding sites between GATA2 and the promoter region of MDM2 predicted on the UCSC website; b, binding relationship between GATA2 and MDM2 validated through the ChIP assay (*p < 0.05, two-way ANOVA); c, MDM2 expression in OS samples predicted on TCGA database (*p < 0.05, unpaired *t* test); d, relevance between MDM2 and prognosis of OS patients predicted on TCGA database (*p < 0.05, Kaplan-Meier analysis); e, a negative correlation between the predicted GATA2 and MDM2 expression in OS samples (*p < 0.05, Pearson’s correlation analysis); f, MDM2 expression in tumor and normal tissues from OS patients determined by RT-qPCR (n = 31, *p < 0.05, paired *t* test); g, relevance between MDM2 expression and the survival rate of the OS patients (*p < 0.05 according to the Kaplan-Meier analysis); h, a positive relevance between GATA2 and MDM2 expression in the tumor tissues from patients (*p < 0.05, Pearson’s correlation analysis); i, MDM2 expression in OS cell lines and in hFOB1.19 cells determined by RT-qPCR (*p < 0.05, one-way ANOVA); j, mRNA expression of MDM2 in cells transfected with MDM2-OE examined by RT-qPCR (*p < 0.05, one-way ANOVA); k, protein level of p53 in MG-63 cells after different treatment detected by western blot analysis (*p < 0.05, one-way ANOVA). Repetition = 3

The above pathway analyses suggested that miR-15a and GATA2 are candidate regulators of the p53 signaling pathway. As previously discussed, MDM2 is a well-established oncogenic E3 ligase that controls the ubiquitination and degradation of p53 and is implicated in OS progression [19], whereas another member of the GATA-binding factor family, GATA4, was reported as a positive regulator of MDM2 [18]. Therefore, we speculated that GATA2 may regulate MDM2 to govern the p53 signaling pathway. According to the prediction on the UCSC website, there are binding sites between GATA2 and the promoter region of MDM2 (Figure 7(a)). The binding relationship between GATA2 and the MDM2 promoter was validated through the ChIP assay. The MDM2 promoter sequence was collected, in which the enrichment of GATA2 was examined. It was observed that exotic overexpression of GATA2 significantly increased the enrichment of GATA2 in the MDM2 promoter (Figure 7(b)), suggesting that GATA2 could specifically bind to MDM2 promoter. The data from TCGA database suggested a high expression profile of MDM2 in patients (Figure 7(c)), indicating a poor prognosis in patients (Figure 7(d)). Furthermore, a positive correlation between GATA2 and MDM2 was confirmed (Figure 7(e)). Thereafter, MDM2 expression was determined in the collected tissue samples. Consequently, increased expression of MDM2 was found in the tumor tissues compared to that in normal tissues (Figure 7(f)). Patients with higher MDM2 expression showed a lower survival rate (Figure 7(g)). However, a positive correlation between GATA2 and MDM2 expression in tumor tissues was observed (Figure 7(h)). In addition, the RT-qPCR results showed that the mRNA expression of MDM2 was decreased in OS cell lines (Figure 7(i)). To further confirm the possible involvement of the p53 signaling pathway in the miR-15a/GATA2/MDM2-mediated events, MDM2 overexpression was introduced into cells (Figure 7(j)). The protein levels of p53 in MG-63 cells transfected with miR-15a mimic, GATA2-OE, MDM2-OE, GATA2-OE + miR-15a, and GATA2-OE + MDM2-OE were examined. It was found that overexpression of MDM2 or/and GATA2 decreased the protein level of p53 in MG-63 cells, whereas upregulation of miR-15a recovered p53 expression. Furthermore, joint overexpression of GATA2 and MDM2 led to a further decline in p53 expression in cells (Figure 7(k)).

### The miR-15a/GATA2/MDM2 axis mediates the growth of OS cells

**Figure 8.**
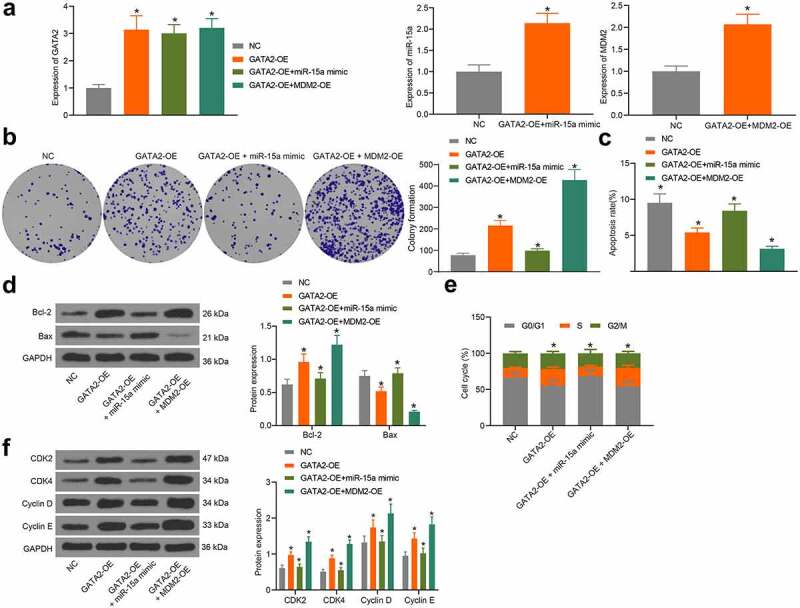
miR-15a promotes apoptosis and cell cycle arrests of OS cells. a, transfection efficiency of GATA2-OE, miR-15a mimic and MDM2-OE in MG-63 cells evaluated by RT-qPCR (*p < 0.05, one-way ANOVA); b, proliferation of cells determined by the colony formation assay (*p < 0.05, one-way ANOVA); c, apoptosis of MG-63 and U2-OS cells determined by flow cytometry (*p < 0.05, one-way ANOVA); d, protein levels of Bcl-2 and Bax in cells determined by western blot analysis (*p < 0.05, two-way ANOVA); e, cell cycle progression in cells determined by flow cytometry (*p < 0.05, two-way ANOVA); f, protein levels of CDK2, CDK4, cyclin D, cyclin E in cells determined by western blot analysis (*p < 0.05, two-way ANOVA). Repetition = 3

To validate the interactions among miR-15a, GATA2, and MDM2, overexpression of GATA2, co-overexpression of miR-15a and GATA2, or co-overexpression of GATA2 and MDM2 was introduced in MG-63 cells, and the transfection efficacy was validated by RT-qPCR (Figure 8(a)). The colony formation assay suggested that the proliferation activity of MG-63 cells was increased upon GATA2 overexpression, whereas this increase was blocked following miR-15a upregulation but further strengthened upon MDM2 overexpression (Figure 8(b)). Flow cytometry suggested that overexpression of GATA2 or MDM2 decreased the number of apoptotic MG-63 cells, whereas miR-15a still increased the apoptosis of cells that was initially reduced by GATA2 (Figure 8(c)). Western blot analysis also showed that the Bax/Bcl-2 ratio in cells was decreased by GATA2 and MDM2, but recovered by miR-15a (Figure 8(d)). Furthermore, flow cytometry results also showed that GATA2 and MDM2 promoted cell cycle progression, whereas miR-15a induced cell cycle arrest at the G0/G1 phase (Figure 8(e)). In addition, western blot analysis suggested that the levels of CDK2, CDK4, cyclin D, and cyclin E in MG-63 cells were increased by GATA2 and MDM2 but suppressed by miR-15a (Figure 8(f)).

## Discussion

With approximately 5 million incidences per year and an unsatisfactory survival rate in affected patients, OS remains a health concern in the young population [27]. Exosomes are emerging noninvasive tools that transport lipids, proteins, and miRNAs or other potential biomarkers for clinical purposes, which have aroused increasing concern in the management of human diseases, including OS [12,28]. Comprehensive bioinformatics analyses are useful and helpful in analyzing gene expression, signaling pathways, and protein functions under specific physiological and pathological conditions [29]. Using integrated bioinformatics analyses and experimental evidence, this study reported that serum-derived exosomal miR-15a suppressed the GATA2/MDM2 axis to inhibit the growth and cell cycle progression of OS cells through the p53 signaling pathway.

Data on the GSE70367 and GSE65071 datasets suggest that miR-15a is a deregulated miRNA in OS samples. The further prediction on TCGA and our RT-qPCR results suggested a poor expression profile of miR-15a in OS tissues and cells cells. In addition, poor expression of miR-15a was found to be associated with poor survival in patients. This was partially consistent with a previous study by Shi *et al*. which suggested that downregulation of miR-15a indicated poor clinical outcome in OS patients [30]. The anti-oncogenic role of the miR-15a-miR16 cluster has been well summarized [31]. In OS cells, miR-15a has been reported to suppress proliferation and metastasis, whereas encouraging apoptosis and cell cycle arrest by binding to different mRNA targets [10,32]. Abnormal cell cycle progression is a hallmark of malignancies, triggering the initiation and development of cancers [33]. In this study, altered expression of miR-15a was further induced in OS cells. Consequently, miR-15a exhibited potent inhibitory effects on cell viability and aggressiveness, while it promoted apoptosis and blocked cell cycle progression. From a molecular perspective, the ratio of Bax/Bcl-2 was increased, whereas the cell cycle marker proteins were suppressed upon miR-15a upregulation. In agreement with the *in vitro* results, upregulation of miR-15a suppressed the growth of xenograft tumors in nude mice.

miR-15 was suggested as an exosomal miRNA according to bioinformatics prediction. The abundance of miR-15a was validated in the extracted serum-derived exosomes. These exosomes were internalized by OS cells. A recent study by Wang *et al*. reported that exosomal miR-21 expression in serum and plasma from patients with OS differed from that in normal patients, and that exosomal miR-21 plays important roles in OS tumorigenesis [34]. In addition, a review by Cuscino *et al*. revealed that eight exosomal miRNAs may be implicated in OS progression [35]. miR-15a-5p has been validated as an exosomal miRNA that is responsible for the reduced proliferation and metastasis of lung cancer A549 cells [36]. This study reported that exosomal miR-15a in OS patients might reduce the malignant development of OS cells; however, the molecules needed further exploration and validation.

GATA2, a target of miR-15a, was subsequently selected as the candidate gene for the following study because it was predicted to be highly expressed in OS samples, and either GATA2 and miR-15a were suggested to be enriched in the p53 signaling pathway. The oncogenic role of GATA2 has been validated in prostate cancer [15], which enhances the invasiveness, dissemination, and survival of cancer cells. In addition, high expression of GATA2 was associated with an increased risk of unfavorable clinical outcomes in patients with prostate cancer after radical prostatectomy [37]. Similarly, GATA2 has also been suggested to be essential for non-small cell lung cancer malignancy driven by the RAS oncogene [38]. This study validated that high expression of GATA2 in OS patients indicated reduced survival time. In tumor cells, overexpression of MDM2 is a major contributor to p53 degradation, in addition to mutation or deletion of the TP53 gene [39]. Therefore, blocking the interaction between MDM2 and p53 or suppressing MDM2 may serve as a therapeutic strategy for cancer treatment [40,41]. In this study, a binding relationship between GATA2 and the promoter region of MDM2 was validated, and a positive correlation between GATA2 and MDM2 was confirmed in the OS samples. Therefore, increased transcription of MDM2 might be responsible for the oncogenic events of GATA2 in OS. To validate this, we further demonstrated that GATA2 and/or MDM2 overexpression suppressed the protein level of p53 in cells, whereas the p53 level was recovered after miR-15a upregulation. The joint experiments also found that overexpression of GATA2 or MDM2 promoted proliferation and cell cycle progression in OS cells. This promotion was blocked following miR-15a upregulation again. However, due to time and funding limitations, the roles of miR-15a inhibition and GATA2/MDM2 silencing on p53 activity in cells were not included in the present study. Moreover, animal studies were not involved as well. We would like to examine the role of exosomal miR-15a and its interaction with the GATA2/MDM2 axis and the p53 signaling pathway in animals in future studies to validate the potential tumor-suppressing role of exosomal miR-15a in OS.

## Conclusion

**Figure 9.**
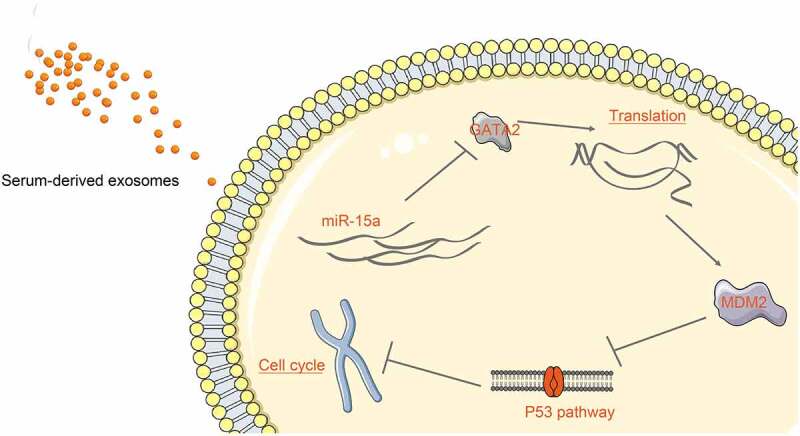
A diagram representation of the molecular mechanism. In OS cells, miR-15a directly binds to GATA2 mRNA, whereas GATA2 promotes MDM2 transcription and suppresses the activity of the p53 signaling pathway. miR-15a suppresses while the GATA2/MDM2 axis promotes proliferation and cell cycle progression of cells. miR-15a is one of the cargos of the serum-derived exosomes and can be delivered to the cells by the exosomes

In conclusion, this study demonstrated that miR-15a may be delivered into OS cells through serum-derived exosomes, and it binds to GATA2 to suppress the MDM2 transcription and reactivate the p53 signaling pathway activity, therefore suppressing cell growth and cell cycle progression *in vitro* (Figure 9). This study may provide a new understanding of the mechanism of action involved in the tumor-suppressive function of miR-15a in OS. However, animal studies are required in the future to validate the mechanism *in vivo*.

## Data Availability

The analyzed data sets generated during the present study areavailable from the corresponding author on reasonable request.
